# Multinuclear Metal-Binding Ability of the N-Terminal Region of Human Copper Transporter Ctr1: Dependence Upon pH and Metal Oxidation State

**DOI:** 10.3389/fmolb.2022.897621

**Published:** 2022-05-05

**Authors:** Maria Incoronata Nardella, Mariagrazia Fortino, Alessandra Barbanente, Giovanni Natile, Adriana Pietropaolo, Fabio Arnesano

**Affiliations:** ^1^ Department of Chemistry, University of Bari Aldo Moro, Bari, Italy; ^2^ Dipartimento di Scienze Della Salute, University of Catanzaro, Catanzaro, Italy

**Keywords:** Ctr1, copper, silver, metal transport, redox processes, NMR specroscopy, mass spectrometry, DFT calculations

## Abstract

The 14mer peptide corresponding to the N-terminal region of human copper transporter Ctr1 was used to investigate the intricate mechanism of metal binding to this plasma membrane permease responsible for copper import in eukaryotic cells. The peptide contains a high-affinity ATCUN Cu(II)/Ni(II)-selective motif, a methionine-only MxMxxM Cu(I)/Ag(I)-selective motif and a double histidine HH(M) motif, which can bind both Cu(II) and Cu(I)/Ag(I) ions. Using a combination of NMR spectroscopy and electrospray mass spectrometry, clear evidence was gained that the Ctr1 peptide, at neutral pH, can bind one or two metal ions in the same or different oxidation states. Addition of ascorbate to a neutral solution containing Ctr1_1-14_ and Cu(II) in 1:1 ratio does not cause an appreciable reduction of Cu(II) to Cu(I), which is indicative of a tight binding of Cu(II) to the ATCUN motif. However, by lowering the pH to 3.5, the Cu(II) ion detaches from the peptide and becomes susceptible to reduction to Cu(I) by ascorbate. It is noteworthy that at low pH, unlike Cu(II), Cu(I) stably binds to methionines of the peptide. This redox reaction could take place in the lumen of acidic organelles after Ctr1 internalization. Unlike Ctr1_1-14_-Cu(II), bimetallic Ctr1_1-14_-2Cu(II) is susceptible to partial reduction by ascorbate at neutral pH, which is indicative of a lower binding affinity of the second Cu(II) ion. The reduced copper remains bound to the peptide, most likely to the HH(M) motif. By lowering the pH to 3.5, Cu(I) shifts from HH(M) to methionine-only coordination, an indication that only the pH-insensitive methionine motif is competent for metal binding at low pH. The easy interconversion of monovalent cations between different coordination modes was supported by DFT calculations.

## Introduction

An elaborate machinery controls the numerous metal ions circulating in the blood and stored in the cells of living organisms. The intracellular concentration of these metal ions is finely regulated because, despite their beneficial effects, they are potentially toxic (particularly those redox active) and can participate in aberrant reactions.

In the case of copper (Cu), the metal participates in various cellular metabolic processes, such as oxidative phosphorylation (Hu et al., 1977; Tsukihara et al., 1995), formation of connective tissue (Kagan and Li, 2003), iron metabolism (Dancis et al., 1994), maturation of neuropeptides (Prigge et al., 2000), detoxification of free radicals (Baker et al., 2017), pigmentation (Matoba et al., 2006), etc. To sustain copper demand, while minimizing the risk of toxic side effects, is critically important for cell viability. For this reason the homeostasis of Cu(I) is finely controlled by a complex protein system, which keeps the metal ion protein-bound while it is delivered to the various intracellular compartments, so preventing the toxic effects stemming from intracellular accumulation of unbound Cu(I) species (O’Halloran and Culotta, 2000; Huffman and O’Halloran, 2001; Field et al., 2002; Boal and Rosenzweig, 2009; Robinson and Winge, 2010; Nevitt et al., 2012; Palumaa, 2013; Lutsenko, 2021). The copper transporter 1 (Ctr1) is a protein essential for copper transport across the plasma membrane (Dancis et al., 1994; Lee et al., 2001). Human Ctr1 (hCtr1) is a membrane protein of approximately 28 kDa and made of 190 amino acids, which features three transmembrane α-helices, a C-terminal domain of 15 amino acids and a long N-terminal extracellular domain (ectodomain) of 67 amino acids (Zhou and Gitschier, 1997; Pope et al., 2012). The exact mechanism by which copper enters the cell is not entirely elucidated, however experimental evidence indicates that three units of Ctr1 are associated to form a pore acting as an ion channel (De Feo et al., 2009; Ren et al., 2019). The localization of Ctr1 is controlled by the intra and extra-cellular copper concentration. An increase in the extracellular concentration of copper induces endocytosis of Ctr1, so reducing the transit of the metal ion across the membrane (Petris et al., 2003; Guo et al., 2004; Van Den Berghe et al., 2007). Conversely, a decrease in copper concentration restores the Ctr1 level at the plasma membrane (Molloy and Kaplan, 2009; Clifford et al., 2016). Proteins homologous to Ctr1 transport selectively the Cu(I) ions, while in the extracellular environment the metal is present mainly in the oxidized Cu(II) form. Therefore, the intervention of reducing agents or enzymes capable of transforming Cu(II) into Cu(I) is required before the metal can be transported into the cytosol. In the case of yeast *S. cerevisiae*, two selective reductases for copper and iron are present (Fre1 and Fre2) (Hassett and Kosman, 1995; Georgatsou et al., 1997). In humans, there is a family of proteins, called STEAP, capable of reducing the two metal ions and facilitate their acquisition by cells (Ohgami et al., 2006; Oosterheert et al., 2018). An alternative, simplified, copper reduction model has also been proposed. It is based on the observation that the N-terminal end of hCtr1 is able to bind and reduce Cu(II) to Cu(I) using ascorbate, and to stabilize Cu(I) thanks to the presence of histidine (His) and methionine (Met) motifs in monomeric (Pushie et al., 2015) or multimeric (Galler et al., 2020) arrangements. The binding of Cu(I) to hCtr1 induces a conformational change, which promotes the interaction of the ectodomain with the cell membrane and may represent the initial step of the Cu(I) uptake process (Yang et al., 2019).

In the present study, we investigated the metal binding properties of a 14mer peptide corresponding to the first 14 amino acids of the N-terminal region of human Ctr1 (Ctr1_1-14_). The peptide contains the amino acid motifs essential for the acquisition of copper from the extracellular environment. The initial H_2_N-X_2_-His motif (known as the ATCUN motif) is also found in human serum albumin (HSA) and is selective for Cu(II) and Ni(II) (Bossak et al., 2018; Gonzalez et al., 2018). Cu(II) binds to this site in a square planar coordination geometry involving four nitrogen atoms (4N): the free NH_2_ terminus, a histidine residue in the third position and the two intervening amidic nitrogens (Santoro et al., 2018).

In the N-terminal region, both hCtr1 and yeast Ctr1 (yCtr1) exhibit Met-rich motifs MxM and MxxM, able to bind Cu(I) (Jiang et al., 2005; Rubino et al., 2010). Furthermore, hCtr1, unlike yCtr1, contains also histidine-rich motifs that have been implicated in the acquisition of Cu(II) and Cu(I) in mammals (Haas et al., 2011). In particular, quite close to the N-terminal end of hCtr1 there is a bis-His (HH) motif (Himes et al., 2007; Shearer and Szalai, 2008) flanked by a Met (the HHM motif) capable of binding and stabilizing Cu(I) (Pushie et al., 2015). The contiguous presence of ATCUN and HHM motifs suggests a possible interaction with copper in both oxidation states (Haas et al., 2011; Pushie et al., 2015).

Based on the above consideration, we deemed it appropriate to investigate the interaction of Ctr1_1-14_ with copper in both oxidation states and also with the redox-inactive Ag(I) ion, as a Cu(I) mimic. Moreover, since the endosomal compartment where hCtr1 is internalized is acidic, the investigation was carried on at different pH values (from neutral to highly acidic).

## Materials and Methods

The 14mer Ctr1_1-14_ peptide (sequence MDHSHHMGMSYMDS, MM: 1665.85 Da; pI: 5.68) was purchased from Genscript Biotech (Piscataway, New Jersey, United States) and used without further purification.

### Nuclear Magnetic Resonance (NMR) Spectroscopy

NMR samples were prepared by dissolving Ctr1_1-14_ in phosphate buffer (Pi, 25 mM, pH = 7 or 3.5) or in 2-(N-morpholino)ethanesulfonic acid (MES, 10 mM, pH = 7) at 250 μM concentration. NMR titrations were performed by gradually adding CuSO_4_ or AgNO_3_ dissolved in pure water to the apopeptide solution, directly in the NMR tube. 1D ^1^H NMR spectra were recorded at 298 K on a Bruker Avance 300 Ultrashield spectrometer equipped with a double resonance broad-band probe with Z-Gradient. 1D ^1^H NMR spectra were acquired with a relaxation delay of 1.5 s, 256 scans, 32K data points and a spectral width of 16 ppm. ^1^H chemical shifts were referenced to trimethylsilylpropanoic acid (TSP, δ_1H_ = 0.0 ppm).

### Electrospray Ionization Mass Spectrometry (ESI-MS)

Solutions of Ctr1_1-14_ (200 μM) and its adducts with Cu(II) and Ag(I), at different metal:peptide ratios, were prepared in NH_4_OAc (20 mM) at 25 °C. Aliquots of these samples were diluted with buffer to a final concentration of 20 μM and directly infused into the mass spectrometer, an Agilent 6530 Accurate-Mass Quadruple Time-of-Flight (Q-TOF) system equipped with an electrospray interface. Ionization was achieved in the positive or negative ion mode by application of 4.0 kV at the entrance of the capillary; the pressure of the nebulizer gas was 20 psi. The drying gas was heated to 325 °C and introduced at a flow rate of 10 μL/min. Full-scan mass spectra were recorded in the mass/charge (*m/z*) range of 50–3,000. Isotopic distributions were calculated with the program Molecular Weight Calculator (https://omics.pnl.gov/software/molecular-weight-calculator).

### Modeling Calculations

The initial coordinates of the Ctr1_1-14_ peptide were obtained using the CHIMERA software (Pettersen et al., 2004). The coordinates were energy minimized through the Steepest Descent Algorithm and equilibrated with 9 ns of standard molecular dynamics at 300 K using the amber99SB force field (Ivani et al., 2016) and the TIP3P potential (Jorgensen et al., 1983) for water molecules, within the GROMACS MD code - version 2020.2 (Abraham and van der Spoel, 2020). Parallel Bias Metadynamics (PBMetaD) (Pfaendtner and Bonomi, 2015) simulations were subsequently performed using the GROMACS MD code - version 2020.2 (Abraham and van der Spoel, 2020) in the NVT ensemble, using the gyration radius as collective variable. Gaussians with initial height equal to 1.2 kJ/mol and width of 0.2 nm were deposited with a bias factor equal to 20. The total time of the simulation was 45 ns.

The three highly populated conformers were extracted from the PBMetaD trajectory through cluster analysis using the method of Daura and van Gunsteren (Daura et al., 1999) and were used as starting coordination geometries. The coordination models of Ctr1_1-14_ with Ag(I) were built considering the residues from Met1 to Met12 and capping the side chains of Asp2, Ser4, Ser10, Tyr11, Asp13 and Ser14 with hydrogen atoms to reduce the computational cost and maintain simulation accuracy. The derived coordinates were optimized at *ab initio* level, within the Density Functional Theory (DFT) framework by using the PBE functional, suitable for this type of systems (Ernzerhof and Perdew, 1998) and the DGDZVP basis sets (Godbout et al., 1992) with Gaussian16 (Frisch et al., 2016) suite of quantum chemical programs. The use of double-zeta DGDZVP basis set allows to combine a high computational speed and a high accuracy for the study of large systems (Siiskonen and Priimagi, 2017).

## Results

### Analysis of Ctr1_1-14_ at Neutral and Acidic pH

The Ctr1_1-14_ apopeptide was initially characterized by electrospray ionization mass spectrometry (ESI-MS) to verify its identity and degree of purity. The ESI-MS spectrum carried out in NH_4_OAc shows two main signals with *m/z* ratios of 833.3012 and 555.8680, associated to the doubly and triply charged species, respectively (Supplementary Figure S1; Table 1).

**TABLE 1:** Stoichiometry of Ctr1_1-14_ complexes with Cu(II) and Ag(I) ions observed by ESI-MS.

Ions	Experimental *m/z* ^a^	Calculated *m/z* ^a^	ΔDa	Error (ppm)
*apo*Ctr1_1-14_				
[Ctr1_1-14_ + 2H ]^2+^	833.3012	833.2998	0.0014	2
[Ctr1_1-14_ + 3H ]^3+^	555.8680	555.8692	−0.0012	−2
Ctr1_1-14_-Cu(II)				
[Ctr1_1-14_ + Cu(II)]^2+^	864.7583	864.7618	−0.0035	−4
[Ctr1_1-14_ + Cu(II) + H]^3+^	576.8399	576.8438	−0.0039	−7
Ctr1_1-14_-2Cu(II)				
[Ctr1_1-14_ + 2Cu(II) − 2H]^2+^	895.2160	895.2238	−0.0078	−9
[Ctr1_1-14_ + 2Cu(II) − H]^3+^	597.1451	597.1518	−0.0067	−11
Ctr1_1-14_-Ag(I)				
[Ctr1_1-14_ + Ag(I) + H]^2+^	887.2496	887.2485	0.0011	1
[Ctr1_1-14_ + Ag(I) + 2H]^3+^	591.8346	591.8349	−0.0003	−1
Ctr1_1-14_-2Ag(I)				
[Ctr1_1-14_ + 2Ag(I)]^2+^	940.1882	940.1971	-0.0089	−9
[Ctr1_1-14_ + 2Ag(I) + H]^3+^	627.1270	627.1340	−0.0070	−11
Ctr1_1-14_-Ag(I),Cu(II)				
[Ctr1_1-14_ + Ag(I)+ Cu(II)−H]^2+^	917.6949	917.7104	−0.0155	−17
[Ctr1_1-14_ + Ag(I)+ Cu(II)]^3+^	612.1314	612.1429	−0.0115	−19

a The reported *m/z* ratios are referred to the prevailing isotopologue in the isotope pattern.

**FIGURE 1:**
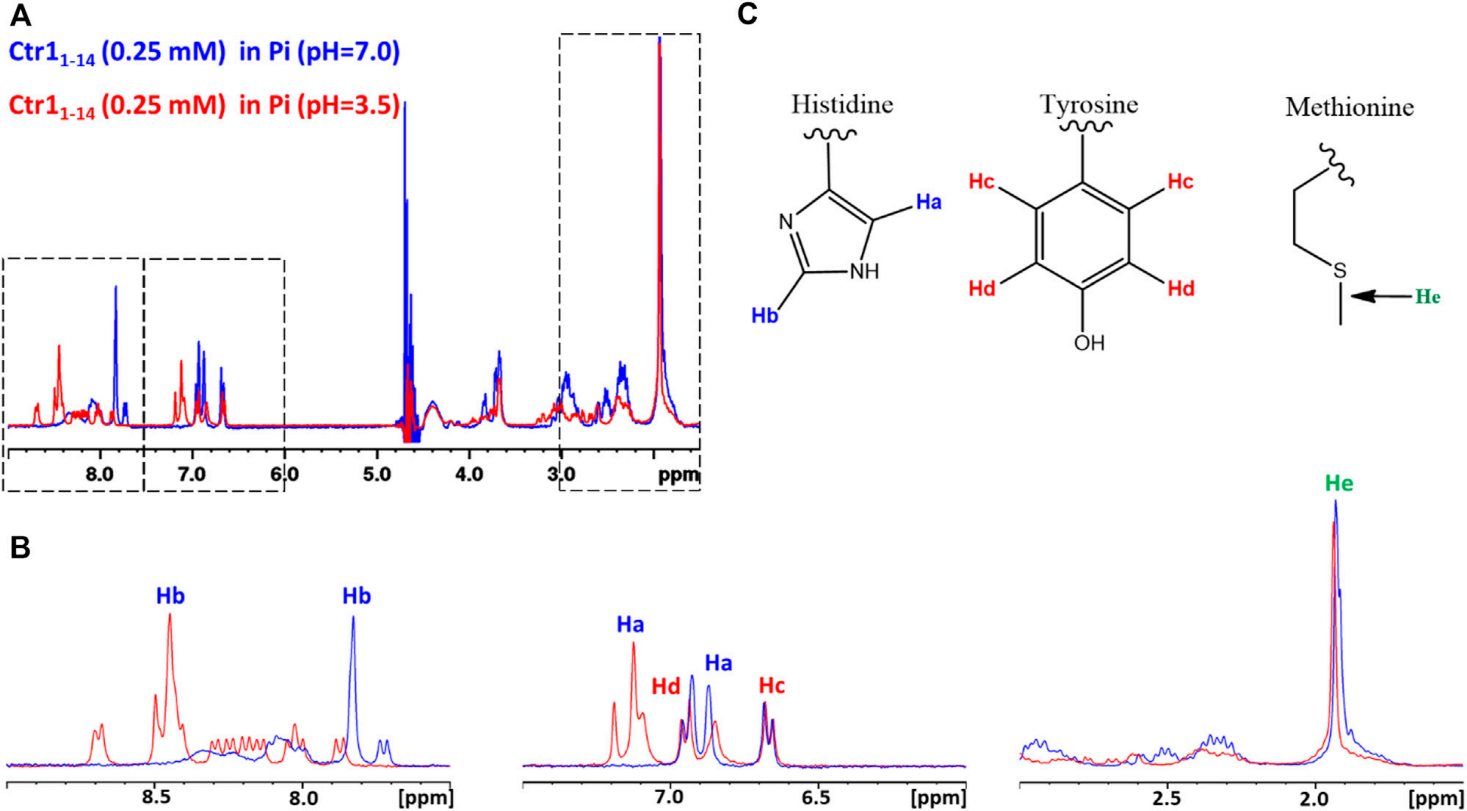
**(A)** Superposition of 1D ^1^H NMR spectra of Ctr1_1-14_ in 25 mM Pi buffer at pH 7.0 (*blue contours*) and 3.5 (*red contours*). The regions enclosed in *dashed boxes* are expanded in **(B)**. The proton assignment scheme is shown in **(C)** where the structure of aminoacid side chains is reported.

Subsequently, a 1D ^1^H NMR spectrum of the apopeptide was recorded in 10 mM MES at pH 7.0 (Supplementary Figure S2) and in 25 mM Pi buffer at pH 7.0 and 3.5 (Figure 1). The main spectral changes between neutral and acidic pH were observed in the region of amides and aromatic protons. While the strong degenerate signal assigned to the ε-CH_3_ methyl protons of the four Met residues and the two signals of Tyr phenyl ring were nearly insensitive to the pH variation; in contrast, the signals of the His imidazole ring were highly perturbed. This result is in agreement with the protonation of imidazole nitrogen (pKa ∼6) in the pH range from 7.0 to 3.5, and suggests that metal ions may prefer different coordination modes depending upon the neutral or acidic environment in which the protein is placed.

**FIGURE 2:**
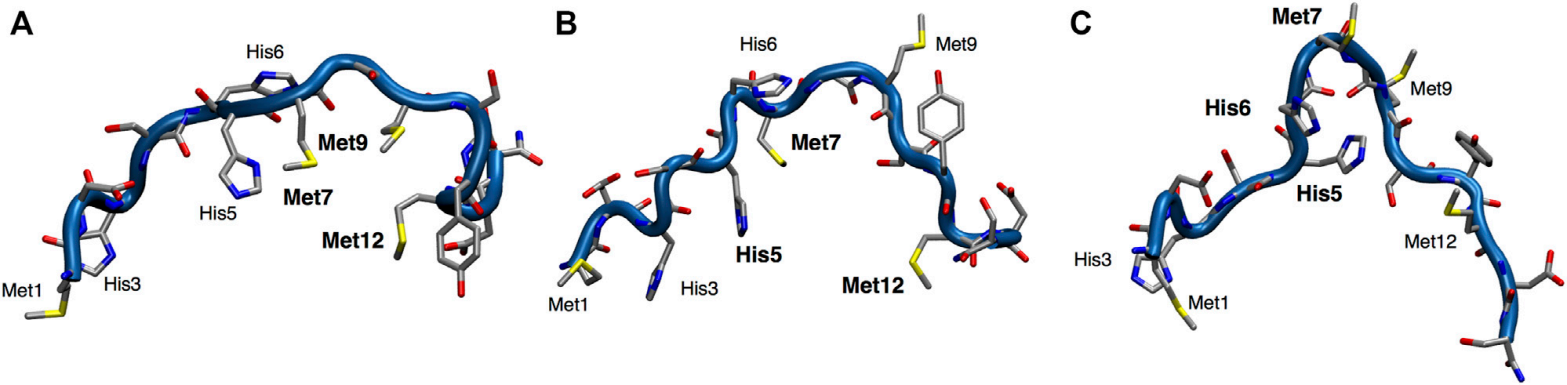
Representation of the three main clusters **(A–C)** extracted from the PBMetaD simulation trajectory. Oxygen is shown in red, nitrogen in blue, carbon in gray and sulfur in yellow.

Structural models of *apo*Ctr1_1-14_ were obtained through Parallel Bias Metadynamics (PBMetaD) (Pfaendtner and Bonomi, 2015) simulations. The main clusters of conformers extracted from the PBMetaD trajectory, representing three possible metal coordination networks, are highlighted in Figure 2. The first cluster is characterized by a close proximity of Met7, Met9 and Met12 and represents a population of 84% (Figure 2A). The second cluster features a network of residues involving His5, Met7 and Met12 and is 10% populated (Figure 2B). Finally, the third cluster features a close proximity of His5, His6 and Met7 and represents a population of 6% (Figure 2C). In the last cluster, a β-turn component of the secondary structure was found between His6 and Met7 residues.

### Interaction of Ctr1_1-14_ With Cu(II) Ions

**FIGURE 3:**
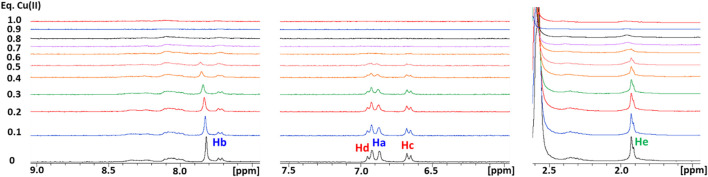
Overlay of three regions of 1D ^1^H NMR spectra of Ctr1_1-14_ in 10 mM MES (pH 7) at different Cu(II):peptide ratios. Proton assignment scheme as shown in Figure 1C.

The interaction between Cu(II) and Ctr1_1-14_ was investigated via NMR by treating the peptide solution (250 μM) with increasing aliquots of a solution of CuSO_4_, up to 1 eq of Cu(II), both in 25 mM Pi and in 10 mM MES buffers at pH 7.0 (Figure 3). In Pi, the Cu(II) ion binds to Ctr1_1-14_ and, given its paramagnetic nature, determines the broadening of the ^1^H NMR signals, in particular those of the His ring which show the greatest relative reduction in intensity. Moreover, the addition of Cu(II) produces only a negligible dipolar hyperfine (pseudocontact) shift, which is consistent with a type-II Cu(II) site (ATCUN) having modest magnetic susceptibility anisotropy (Arnesano et al., 2005). In MES, the broadening of the peptide signals was larger than that observed in the titration carried out in Pi buffer. This can be explained with the poorer coordinating ability of MES with respect to phosphate, which makes more Cu(II) available for binding to the peptide.

**FIGURE 4:**
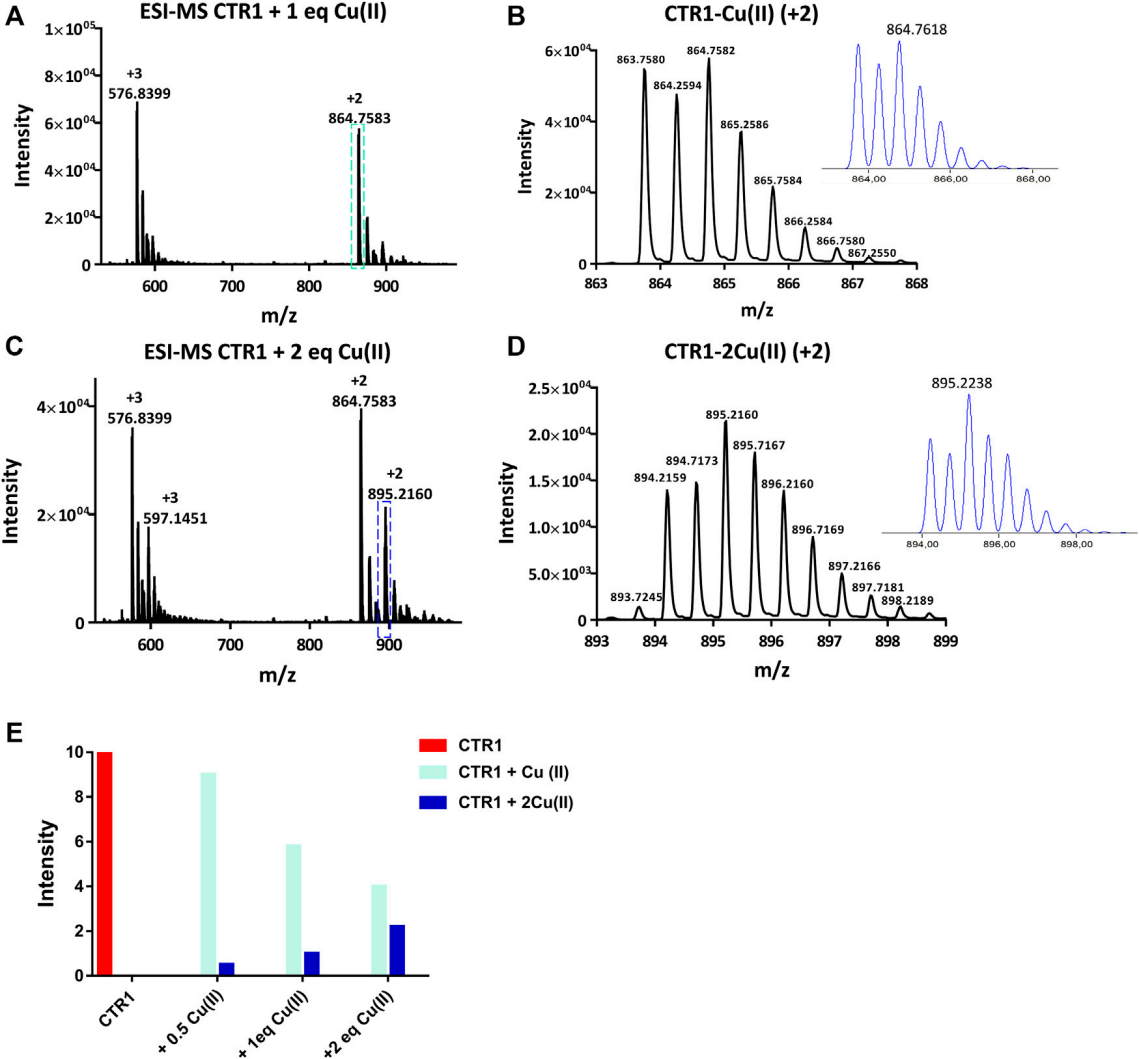
ESI-MS spectra of Ctr1_1-14_ treated with 1 and 2 eq of CuSO_4_ **(A,C)**and magnification of the doubly charged peaks (*cyan and blue dashed boxes*) displayed in **(B,D)**, respectively; the insets show the calculated isotopic distributions. **(E)** Intensity of doubly charged ESI-MS peaks of different Ctr1_1-14_ species as a function of Cu(II):peptide ratio.

The interaction between Cu(II) and the peptide was also monitored by ESI-MS. Two signals were observed at 864.7583 and 576.8399 m*/z*, corresponding to the adduct Ctr1_1-14_-Cu(II) having 2+ and 3 + overall charge, respectively (Figures 4A, B). The addition of Cu(II) beyond one equivalent leads to the almost complete disappearance of the NMR signals, however the ESI-MS spectra indicate the binding of a second Cu(II) ion to the peptide, as deduced from the appearance of two signals at 895.2160 and 597.1451 m*/z* (2+ and 3 + charged Ctr1_1-14_-2Cu(II) adduct) with the predicted isotopic distribution pattern (Figures 4C, D). The relative abundance of Ctr1_1-14_ species (apopeptide and metal adducts) based on ESI-MS peak intensity at different Cu(II):peptide ratios is reported in panel E of Figure 4.

### Interaction of Ctr1_1-14_ With Ag(I) Ions

**FIGURE 5:**
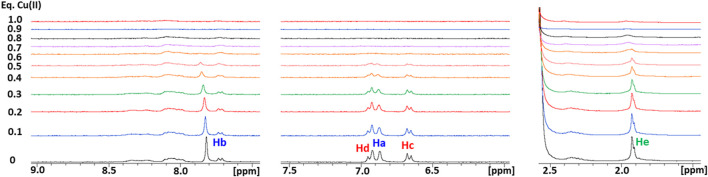
Overlay of three regions of 1D ^1^H NMR spectra of Ctr1_1-14_ in 10 mM MES (pH 7) at different Ag(I):peptide ratios. Proton assignment scheme as shown in Figure 1C.

**FIGURE 6:**
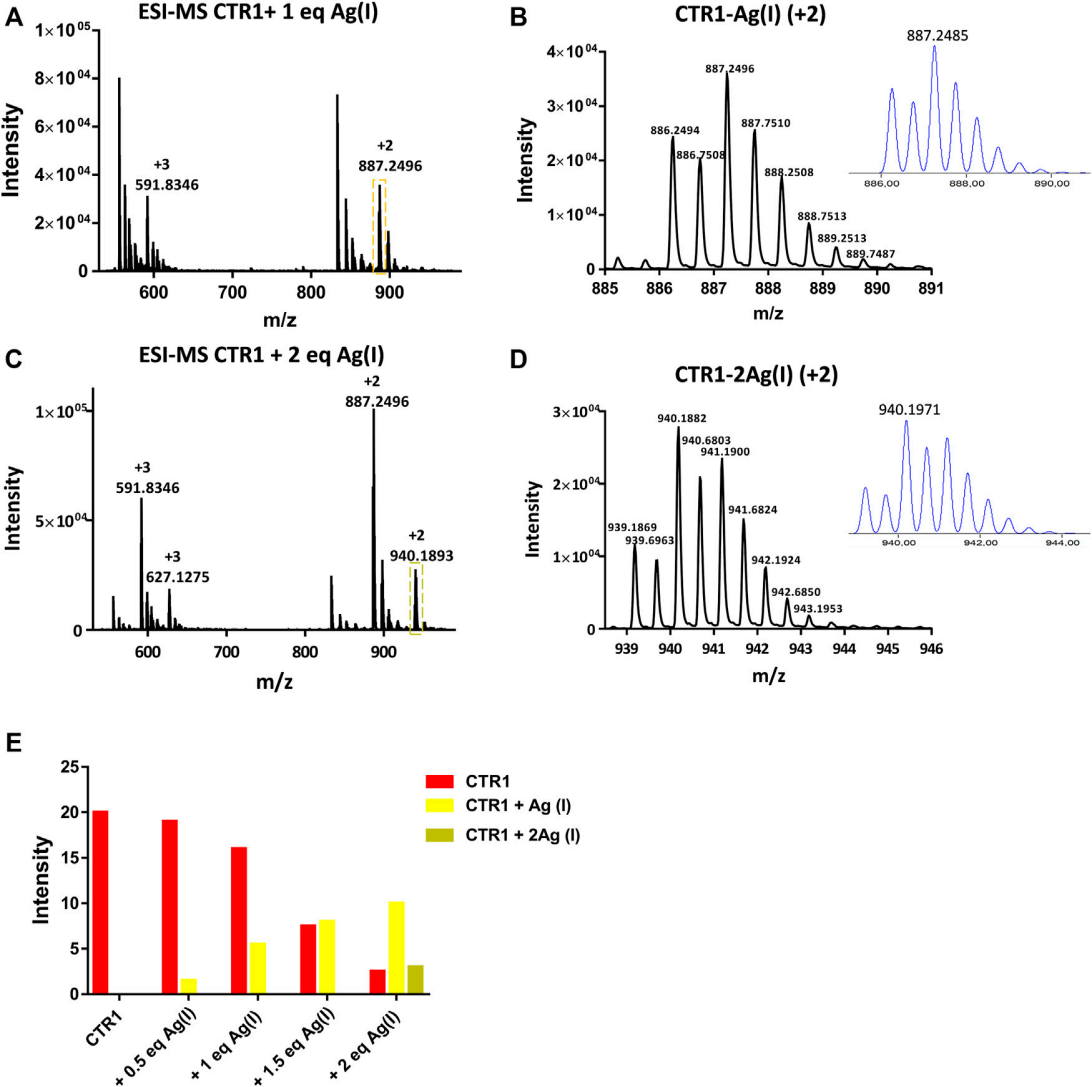
ESI-MS spectra of Ctr1_1-14_ treated with 1 and 2 eq of AgNO_3_ **(A,C)** and magnification of the doubly charged peaks (*yellow and green dashed boxes*) displayed in **(B,D)**, respectively; the insets show the calculated isotopic distributions. **(E)** Intensity of doubly charged ESI-MS peaks of different Ctr1_1-14_ species as a function of Ag(I):peptide ratio.

As a next step, the interaction of Ctr1_1-14_ with redox-stable Ag(I), as a mimic of Cu(I), was investigated via NMR. Thus, a peptide solution (250 μM) was treated with increasing aliquots of AgNO_3_ both in Pi buffer (25 mM) and in MES (10 mM) at pH 7.0. NMR is more informative in the present case given the diamagnetic nature of the metal ion. Ag(I) was found to perturb the signals of His and Met, indicating the involvement of both types of residues in Ag(I) coordination. In particular, the methyl signals of methionines underwent a downfield shift with respect to the apopeptide and a splitting into four peaks, indicating chemical inequivalence of ε-CH_3_ groups. Similarly to Cu(II), also in this case a greater variation of chemical shifts was observed in MES buffer than in Pi. Chemical shift changes were complete after addition of 2 eq of Ag(I) to the peptide (Figure 5). Consistently, the ESI-MS spectra indicated the binding of one and two Ag(I) ions, giving rise to two pairs of signals at 887.2496 and 591.8346 m*/z* and at 940.1882 and 627.1270 m*/z*, respectively (in each pair the first signal belongs to the 2 + fragment and the second to the 3 + fragment; Figures 6A–E).

**FIGURE 7:**
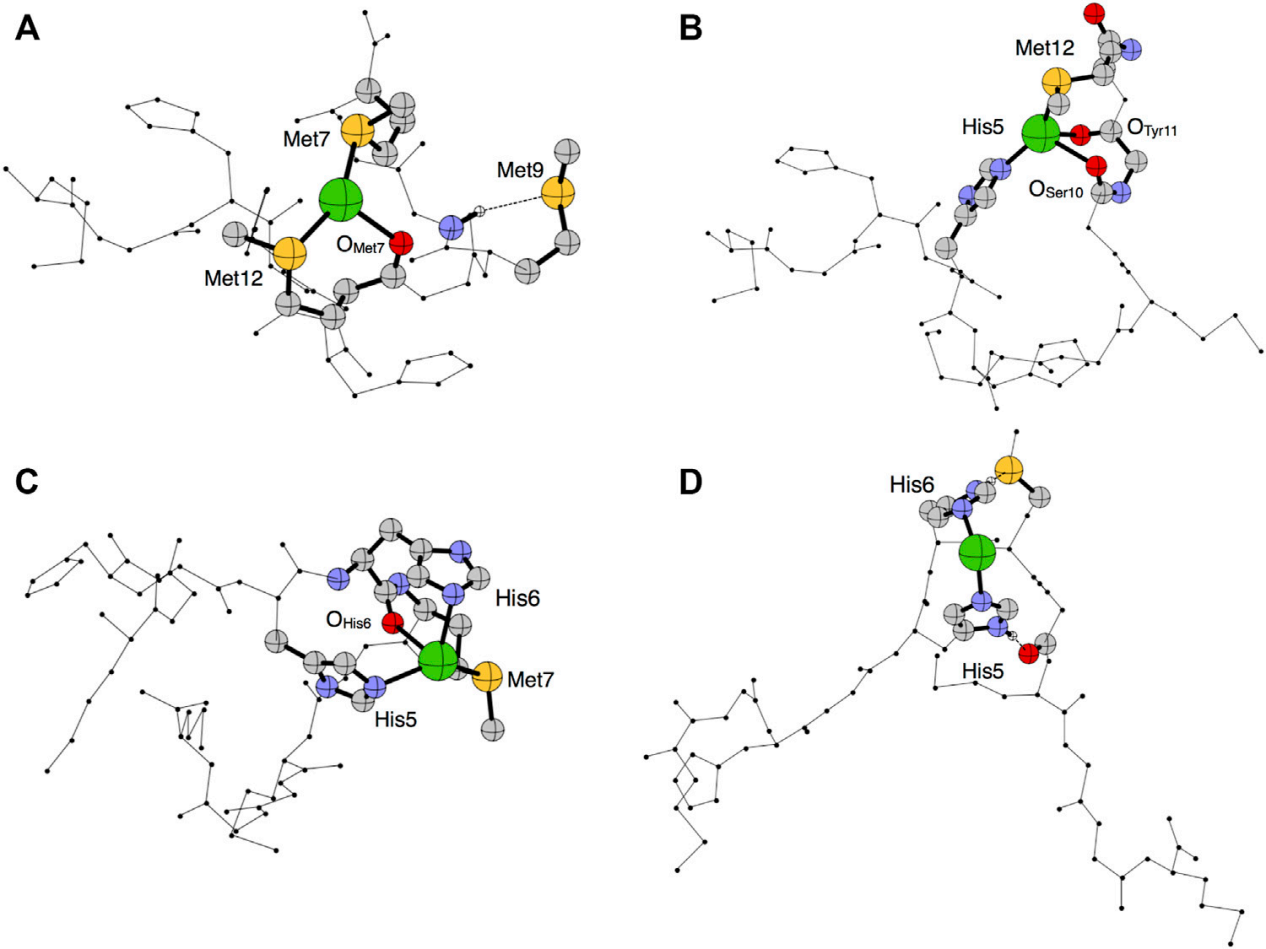
Optimized structural models 1–4 **(A–D)** predicted in the PBE/DGDZVP framework for Ag(I)-Ctr1_1-14_. The silver ion is shown in green, oxygen in red, nitrogen in blue, carbon in gray and sulfur in yellow.

To model the putative metal binding site(s), the Ag(I) ion was coordinated to the network topologies calculated for the apopeptide (see above), and the metal-bound coordinates were optimized at the Density Functional Theory (DFT) level using PBE as density functional (Ernzerhof and Perdew, 1998) and DGDZVP as basis sets (Godbout et al., 1992). The DFT optimized structures of Ctr1_1-14_ coordinated to Ag(I) are reported in Figure 7 and the coordination distances are listed in Table 2.

**TABLE 2:** Coordination parameters derived from the optimized coordinates in gas phase. Distances are reported interatomic distances (in Angstroms) and angles (in degrees) are defined by the atom names (peptide residues are indicated in brackets).

Distances (Å)				Angles (^°^)			
**Model 1**							
** (Met7)Sδ-Ag(I)**	**(Met12)Sδ-Ag(I)**	**(Met7)O-Ag(I)**		**(Met7)Sδ-Ag(I)-(Met12)Sδ**	**(Met7)Sδ-Ag(I)-(Met7)O**	**(Met12)Sδ-Ag(I)-(Met7)O**	
2.57	2.58	2.43		153.7	98.1	105.1	
**Model 2**							
**(His5)Nε2-Ag(I)**	**(Met12)Sδ-Ag(I)**	**(Ser10)O-Ag(I)**	**(Tyr11)O-Ag(I)**	**(His5)Nε2-Ag(I)-(Met12)Sδ**	**(His5)Nε2-Ag(I)-(Ser10)O**	(**Met12)Sδ-Ag(I)-(Tyr11)O**	**(Ser10)O-Ag(I)-(Tyr11)O**
2.27	2.57	2.58	2.53	149.7	112.1	110.3	72.4
**Model 3**							
**(His5)Nε2-Ag(I)**	**(His6)Nε2-Ag(I)**	**(Met7)Sδ-Ag(I)**	**(His6)O-Ag(I)**	**(His5)Nε2-Ag(I)-(His6)Nε2**	**(His6)Nε2-Ag(I)-(Met7)Sδ**	**(Met7)Sδ-Ag(I)-(His6)O**	**(His6)O-Ag(I)-(His5)Nε2**-
2.34	2.45	2.66	2.94	121.5	107.1	82.8	74.9
**Model 4**							
**(His5)Nε2-Ag(I)**	**(His6)Nε2-Ag(I)**			**(His5)Nε2-Ag(I)-(His6)Nε2**			
2.26	2.27			115.0			

Model 1 shows a quasi-trigonal geometry around the Ag(I) ion which involves the sulfur atoms of Met7 and Met12 and the carbonyl of Met7, with a predicted hydrogen bond involving the amide proton and the sulfur atom of Met9 (it can be noted that this model alone could account for different chemical shifts for all methionine methyls). In Model 2, Ag(I) has a pseudo-tetrahedral geometry involving the Nε2 of His5, the sulfur atom of Met12 and the two carbonyl groups of Ser10 and Tyr11. Model 3 is compatible with a quasi-tetrahedral geometry around the Ag(I) ion involving the Nε2 of His5 and His6, the sulfur atom of Met7 and the carbonyl group of His6. In Model 4, Ag(I) has a pseudo-linear coordination geometry involving Nε2 of His5 and His6, eventually bent by the hydrogen bonds between the imidazole ring proton HNδ1 of His6 and the sulfur atom of Met7 and between HNδ1 of His5 and the carbonyl oxygen of Ser10.

The calculated total energy differences are reported in Table 3 and indicate that Model 1 is the lowest in energy, followed by Model 2 and Model 3 with energy differences of 0.47 and 0.82 kcal/mol, respectively. These small energy differences are easily accessible owing to thermal effects at room temperature and suggest a possible interconversion between different coordination modes. Model 4 is predicted to be the highest in energy (ΔE = +6.33 kcal/mol).

**TABLE 3:** PBE/DGDZVP relative energies computed for all investigated models in gas phase.

	|ΔE|(kcal/mol)
Model 1	0.00
Model 2	0.47
Model 3	0.82
Model 4	6.33

### Simultaneous Binding of Cu(II) and Ag(I) to Ctr1_1-14_

Given the presence of multiple metal binding sites in Ctr1_1-14_, the possible interaction of the peptide with both Cu(II) and Ag(I) was investigated.

**FIGURE 8:**
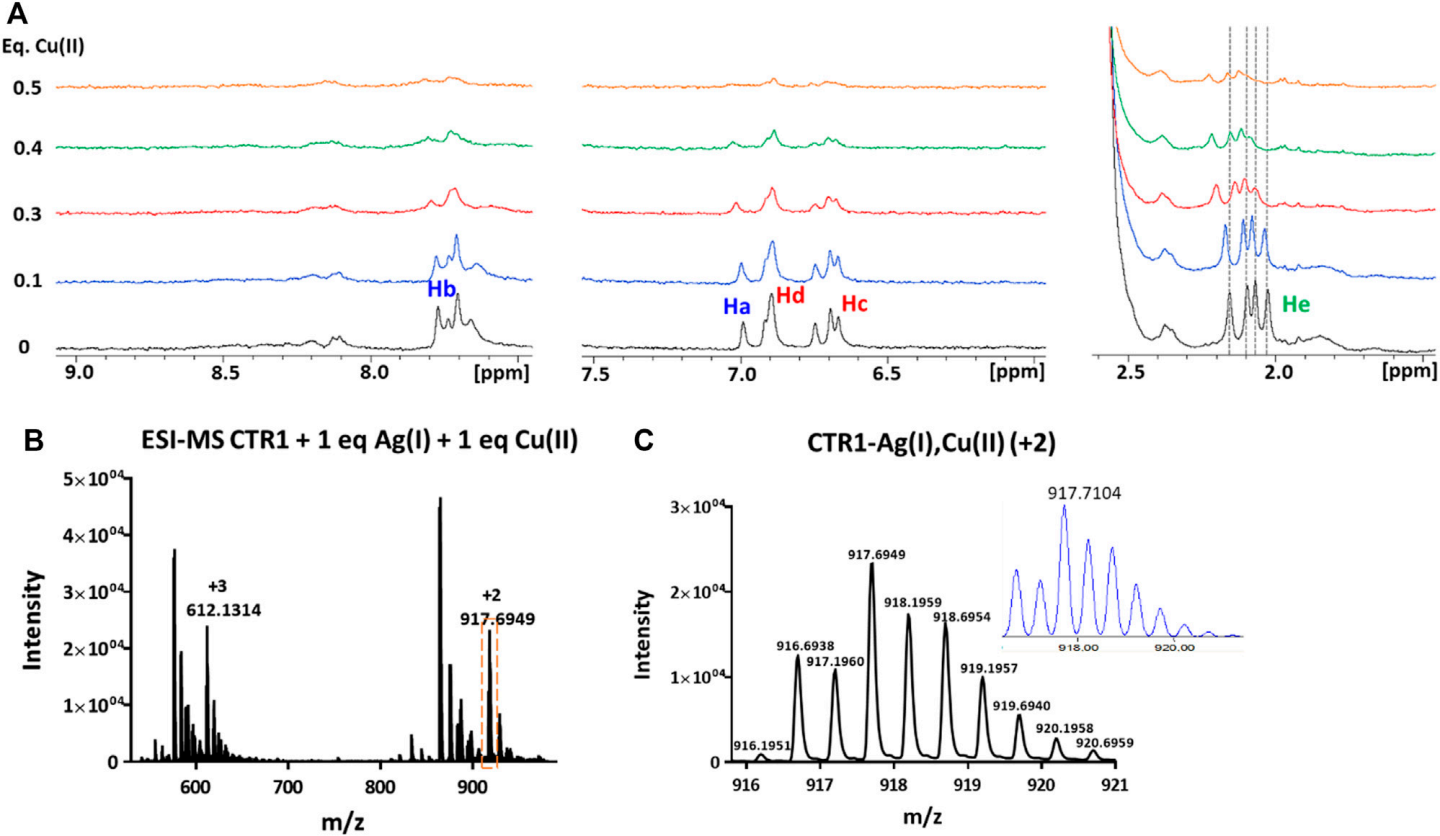
**(A)** Overlay of three regions of 1D ^1^H NMR spectra of Ctr1_1-14_ containing 1 eq of Ag(I) in 10 mM MES (pH 7) treated with Cu(II) at different copper:peptide ratios; the dashed lines indicate the positions of the four methyl signals of methionines (He) before addition of Cu(II). Proton assignment scheme as shown in Figure 1C. **(B)** ESI-MS spectra of Ctr1_1-14_ after treatment with 1 eq of AgNO_3_ and 1 eq of CuSO_4_ and **(C)** magnification of the doubly charged peak (*orange dashed box in B*); the inset shows the calculated isotope distribution.

Addition of Cu(II) to a solution of Ctr1_1-14_ containing 1 eq of Ag(I) in MES (pH 7.0), was found to cause a widespread broadening of the 1D ^1^H NMR signals; however, at sub-stoichiometric Cu(II) addition a shift to lower fields of the methyl signals of methionines could be detected (Figure 8). Since, apart from broadening, Cu(II) alone did not cause any significant shift of apopeptide peaks (see Figure 3), the downfield shift of the ε-CH_3_ signals could indicate a further shift of Ag(I) coordination towards the methionines caused by the interaction of Cu(II) with histidines, whose signals undergo the largest line broadening due to the paramagnetic effect of Cu(II). It is concluded that Ctr1_1-14_ can accommodate a Cu(II) (ATCUN site) and an Ag(I) ion. On the basis of the different Ag(I) coordination models discussed above and displayed in Figure 7, it can be proposed that the conformer with Met-only coordination (Model 1 in Figure 7A), which was already found to be the most favored in the interaction with the free peptide, can become even more populated after addition of Cu(II).

The formation of a heterobimetallic complex of Ctr1_1-14_ with Cu(II) and Ag(I) was confirmed by the ESI-MS spectrum recorded in NH_4_OAc, which shows, after addition of 1 eq of CuSO_4_ to the solution of Ctr1_1-14_ containing 1 eq of AgNO_3_ in MES (pH 7.0), a pair of signals at 917.6949 and 612.1314 m*/z* (2+ and 3 + fragments, respectively), corresponding to the adduct of Ctr1_1-14_ with both Ag(I) and Cu(II), as also confirmed by the full agreement between the experimental and the theoretical isotopic distribution patterns (Figure 8). A similar result was obtained when the order of salt addition was reversed (addition of AgNO_3_ to a solution of Ctr1_1-14_ containing 1 eq of CuSO_4_).

### Interaction of Ctr1_1-14_ With Metal Ions at Low pH

**FIGURE 9:**
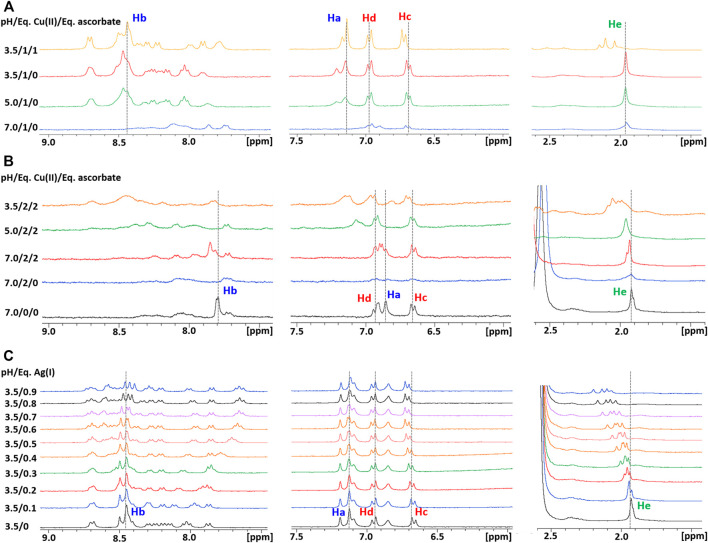
**(A)** Overlay of three regions of 1D ^1^H NMR spectra of Ctr1_1-14_ containing 1 eq of Cu(II) in 25 mM Pi buffer at different pH values, with or without ascorbate; the dashed lines indicate the positions of the corresponding proton signals at pH 3.5 in the absence of ascorbate. **(B)** Overlay of three regions of 1D ^1^H NMR spectra of Ctr1_1-14_ containing 2 eq of Cu(II) in 10 mM MES at different pH values, with or without ascorbate; the dashed lines indicate the positions of the corresponding proton signals at pH 7.0 in the absence of Cu(II). **(C)** Overlay of three regions of 1D ^1^H NMR spectra of Ctr1_1-14_ in 25 mM Pi buffer (pH 3.5) at different Ag(I):peptide ratios; the dashed lines indicate the positions of the corresponding proton signals in the absence of Ag(I).

In accord with the species distribution diagram of the Cu(II) complexes with Ctr1_1-14_, indicating that the affinity of Cu(II) for the peptide drops at low pH due to α-NH_2_ and His protonation (Magrì et al., 2022), the NMR spectra of Ctr1_1-14_ containing 1 eq of Cu(II) in 25 mM Pi buffer show almost complete recovery of the free peptide signals when the pH is lowered from 7.0 to 3.5 (Figure 9A). The ESI-MS spectra at low pH of a solution of Ctr1_1-14_ containing 1 eq of CuSO_4_ confirmed that the Cu(II) ion is detached and only the apopeptide is present (Supplementary Figure S3).

Unlike Cu(II), Ag(I) remains coordinated to Ctr1_1-14_ also at low pH. This was shown by titration of *apo*Ctr1_1-14_ with AgNO_3_ in Pi buffer at pH 3.5. In this acidic environment, Ag(I) addition causes shift and splitting of the ε-CH_3_ signals of methionines, while the His signals remain unperturbed, consistent with the imidazole nitrogens remaining protonated. It was observed also a distinct shift of the tyrosine ring protons (Hc), which may reflect the formation of a macrochelate (including Tyr11) consequent to the binding of Ag(I) to methionines (Figure 9C). The binding of Ag(I) to the peptide at acidic pH was confirmed by the ESI-MS experiment performed on a solution of Ctr1_1-14_ containing 1 eq of AgNO_3_ at pH 3.5. The observation of the pair of signals at 887.2496 and 591.8346 m/z indicates the presence of the Ctr1_1-14_-Ag(I) adduct (2+ and 3+ charged fragments, respectively; Supplementary Figure S3).

### Susceptibility of Cu(II) to Undergo Reduction by Ascorbate

Addition of ascorbate to a neutral solution containing Cu(II) and Ctr1_1-14_ in 1:1 ratio does not cause an appreciable reduction of Cu(II) to Cu(I); in contrast, under acidic conditions (pH 3.5), Cu(II) can be fully reduced by addition of a stoichiometric amount of ascorbate. The formation of Cu(I), consequent to the addition of ascorbate, causes a splitting of the initially degenerate signals of the four ε-CH_3_ groups of methionines (all resonating at ∼1.95 ppm in *apo*Ctr1_1-14_ both under neutral and acidic conditions) into three peaks that are shifted to lower field and integrate for 3, 6, and 3H, respectively. Furthermore, the proton signals of the imidazoles do not undergo any change upon addition of ascorbate, indicating that His residues are not involved in Cu(I) binding at low pH. It can be concluded that, at pH 3.5, the formed Cu(I) ion coordinates to the sulfur atoms of methionines, as already observed for Ag(I).

In a final experiment, Ctr1_1-14_ was loaded with two equivalents of Cu(II) ions in MES at pH 7.0 and then treated with ascorbate. In this case, ascorbate was able to reduce some Cu(II) to Cu(I), even at neutral pH, causing a partial recovery in intensity of the NMR peaks, which showed a downfield shift of His aromatic signals not observed in the solution of Ctr1_1-14_ containing 1 eq of Cu(II) at pH 7.0. When the pH was lowered from 7.0 to 3.5, the ε-CH_3_ signals of Met residues underwent a downfield shift indicative of Cu(I) switching from His to Met coordination (Figure 9B). It can be concluded that the second Cu(II) is more loosely bound to the peptide and can be reduced to Cu(I) by ascorbate at neutral pH and the formed monovalent copper ion coordinates to histidines at neutral pH and to methionines at low pH.

Concerning similarities and differences between Cu(I) and Ag(I) binding to Ctr1_1-14_, information can be drawn from the following observations. At pH 3.5, comparison between the ^1^H NMR spectra of 1:1 complexes of Ctr1_1-14_ with Cu(I) and Ag(I) (top spectra of Figures 9A, C) indicates a more pronounced downfield shift of methionine-methyl signals in the case of Ag(I) with respect to Cu(I). At neutral pH, the comparison between the spectra of Ctr1_1-14_ with Cu(I) and Ag(I) (middle spectra of Figure 9B; Figure 5) suggests that while Cu(I) preferentially binds to the bis-His motif, Ag(I) binds also to Met residues. Both observations are consistent with the softer character of Ag(I), which causes this metal ion to prefer coordination to sulfur over nitrogen.

## Conclusion

The present investigation highlights the ability of the first 14 residues of hCtr1 to host both divalent and monovalent metal ions and to form, beyond monometallic species, also bimetallic complexes, such as Ctr1_1-14_-2Cu(II) and Ctr1_1-14_-2Ag(I).

Regarding the relative affinity of Cu(II) and Ag(I) ions for the peptide under neutral conditions, the ESI-MS peak intensity diagrams (Figure 4E; Figure 6E) indicate that, while the apopeptide peaks disappear after addition of 1 eq of Cu(II), the apopeptide is still the prevailing species after addition of 1 eq of Ag(I). Although, in general, the intensity of the ESI-MS peaks cannot be taken as a measure of the abundance of species in solution, in the present case the observed trend in intensities of the ESI-MS peaks parallels the relative abundances observed in a potentiometric investigation of Ctr1_1-14_ solutions treated with either Cu(II) or Ag(I) ions (Magrì et al., 2022). Moreover, the results are in agreement with isothermal calorimetry data for the titration with Cu(II) or Ag(I) of the entire hCtr1 ectodomain (Ctr1_1-55_) (Du et al., 2013).

By lowering the pH, the Cu(II) ion detaches from the peptide; in contrast the Ag(I) complex can survive in acidic conditions (pH 3.5), but shifts from histidines to methionine-only coordination.

Addition of ascorbate to a neutral solution containing Cu(II) and Ctr1_1-14_ in 1:1 ratio does not cause an appreciable reduction of Cu(II) to Cu(I), indicating a tight binding of Cu(II) to the peptide at the ATCUN site. However, by lowering the pH to 3.5, the Cu(II) detaches from the peptide (as discussed above) and becomes susceptible to reduction to Cu(I) by ascorbate. It is noteworthy that, unlike Cu(II), Cu(I) remains coordinated to the peptide (NMR shift and splitting of the methyls of methionines), possibly by adopting a ligand coordination similar to that observed for Ag(I) at low pH and involving only Met residues (Ag(I) mode of coordination also supported by DFT calculations; Figure 7).

As stated above, Ctr1_1-14_ can also bind two Cu(II) ions, but these Cu(II) ions proved to have different susceptibility to reduction by ascorbate at neutral pH. Therefore, addition of ascorbate to Ctr1_1-14_-2Cu(II) at pH 7.0 causes a partial recovery in intensity of the NMR signals (otherwise heavily broadened by paramagnetic Cu(II)) accompanied by an evident shift of His signals (as compared to the apopeptide) with only a slight variation of Met signals, which is indicative of Cu(I) binding to the HH(M) site. By lowering the pH to 3.5, all Met signals undergo a downfield shift, in accord with Cu(I) shifting from HH(M) to Met-only coordination (and the detachment of the Cu(II) ion). This pH-regulated ligand switching is similar to that previously observed for the lumenal HM loop of ATP7A, a human Cu(I)-transporting ATPase that shuttles between the Golgi organelle and the plasma membrane (Kline et al., 2016).

That at neutral pH the N-terminal region of hCtr1 can simultaneously host a Cu(II) and a Cu(I) ion at different sites is also supported by the observation that a heterobimetallic complex (Ctr1_1-14_-Cu(II),Ag(I)) can be obtained by addition of Ag(I) (a mimic of Cu(I)) to a solution of Ctr1_1-14_ already loaded with Cu(II). Interestingly, the same product is obtained by addition of Cu(II) to a solution of Ctr1_1-14_ loaded with Ag(I).

## Data Availability

The original contributions presented in the study are included in the article/Supplementary Material, further inquiries can be directed to the corresponding author.
