# Supplementary material for: Multinuclear Metal-Binding Ability of the N-Terminal Region of Human Copper Transporter Ctr1: Dependence Upon pH and Metal Oxidation State
Source: Front Mol Biosci. 2022 May 5;9:897621. doi: 10.3389/fmolb.2022.897621 (PMC9117721; doi:10.3389/fmolb.2022.897621)
Supplement: Supplementary file 1 [file Image1.PDF]

Supplementary Material

1 Supplementary Figures

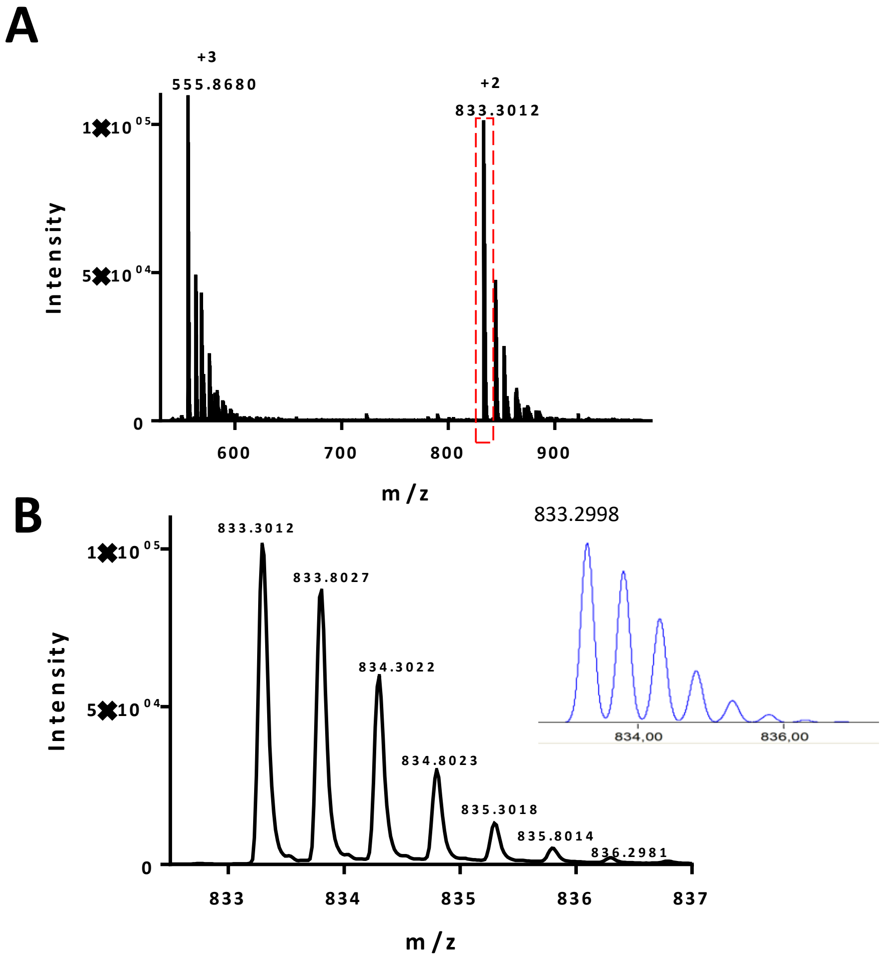

Figure S1. ESI-MS spectrum of Ctr1$_{1\text{-}14}$ apopeptide (A) and magnification of the doubly charged peak (*red dashed box*) shown in (B); the inset shows the calculated isotope distribution.

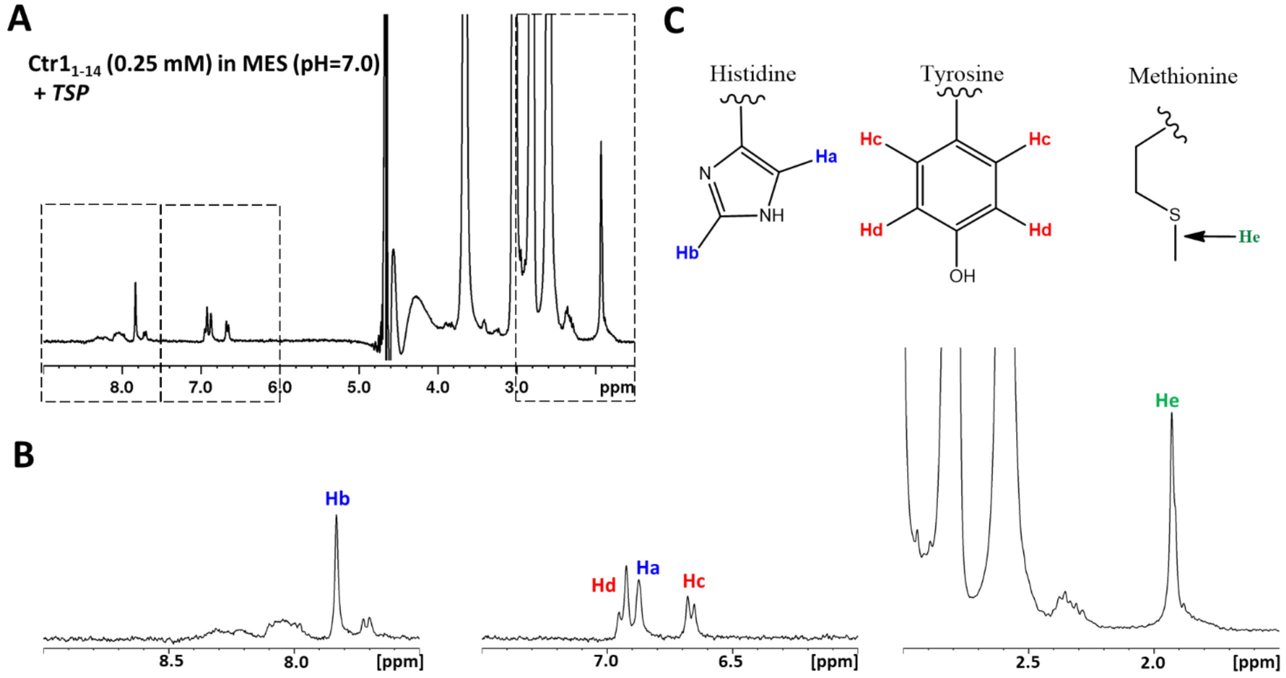

Figure S2. 1D ^{1}H NMR spectra of Ctr1$_{1\text{-}14}$ in 10 mM MES buffer at pH 7.0 (A). The regions enclosed in *dashed boxes* are expanded in (B). The proton assignment scheme is shown in (C) where the structure of aminoacid side chains is reported. *TSP* = trimethylsilylpropanoic acid.

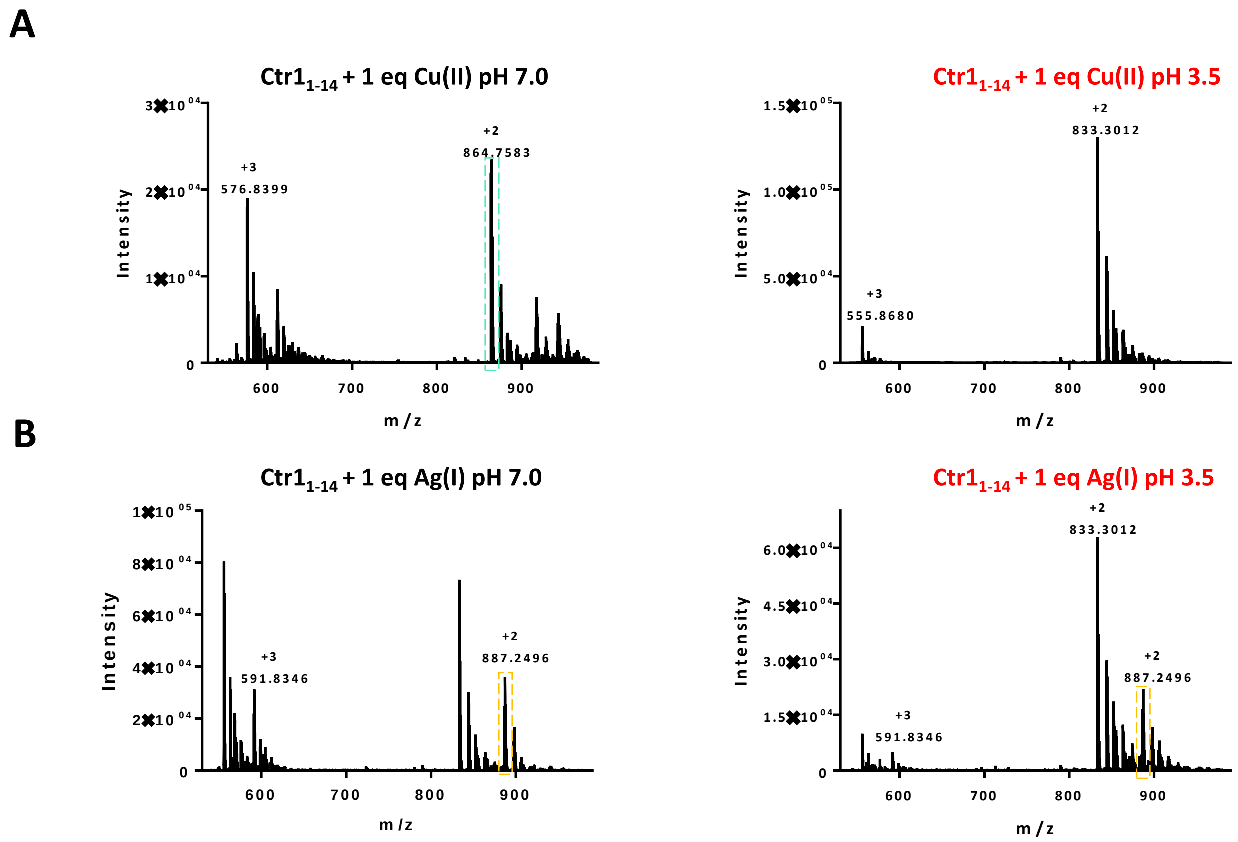

Figure S3. ESI-MS spectra of Ctr1$_{1-14}$ treated with 1 eq of $CuSO_4$ (A) or 1 eq of $AgNO_3$ (B) at pH 7.0 or 3.5. The *cyan* and *yellow dashed boxes* indicate the doubly charged peaks corresponding to Ctr1$_{1-14}$-Cu(II) and Ctr1$_{1-14}$-Ag(I) complexes, respectively.
